# DCE-MRI in Glioma, Infiltration Zone and Healthy Brain to Assess Angiogenesis: A Biopsy Study

**DOI:** 10.1007/s00062-021-01015-3

**Published:** 2021-04-26

**Authors:** Vera C. Keil, Gerrit H. Gielen, Bogdan Pintea, Peter Baumgarten, Angeliki Datsi, Kanishka Hittatiya, Matthias Simon, Elke Hattingen

**Affiliations:** 1 Department of Neuroradiology, University Hospital Bonn, Venusberg-Campus 1, 53127 Bonn, Germany; 2 Department of Radiology, Amsterdam University Medical Center, location VUmc, De Boelelaan 1117, 1081 HV Amsterdam, The Netherlands; 3 Department of Neuropathology, University Hospital Bonn, Venusberg-Campus 1, 53127 Bonn, Germany; 4 Department of Neurosurgery, University Hospital BG Bergmannsheil, Bürkle-de-la-Camp-Platz 1, 44789 Bochum, Germany; 5 Department of Neurosurgery, University Hospital Bonn, Venusberg-Campus 1, 53127 Bonn, Germany; 6 Department of Neurosurgery, University Hospital Frankfurt, Schleusenweg 2-16, 60528 Frankfurt am Main, Germany; 7 Institute of Neuropathology (Edinger Institute), University Hospital Frankfurt, Schleusenweg 2–16, 60528 Frankfurt am Main, Germany; 8 ITZ, Heinrich-Heine-University Düsseldorf, Moorenstraße 5, 40225 Düsseldorf, Germany; 9 Center for Pathology, University Hospital Bonn, Venusberg-Campus 1, 53127 Bonn, Germany; 10 Department of Neurosurgery, Ev. Krankenhaus Bielefeld, Haus Gilead I, Burgsteig 13, 33617 Bielefeld, Germany; 11 Department of Neuroradiology, University Hospital Frankfurt, Schleusenweg 2–16, 60528 Frankfurt am Main, Germany

**Keywords:** Glioma, Biopsy, VEGF, Blood-brain barrier, Multiparametric magnetic resonance imaging

## Abstract

**Purpose:**

To explore the focal predictability of vascular growth factor expression and neovascularization using dynamic contrast-enhanced magnetic resonance imaging (DCE-MRI) in glioma.

**Methods:**

120 brain biopsies were taken in vital tumor, infiltration zone and normal brain tissue of 30 glioma patients: 17 IDH(isocitrate dehydrogenase)-wildtype glioblastoma (GBM), 1 IDH-wildtype astrocytoma °III (together prognostic group 1), 3 IDH-mutated GBM (group 2), 3 anaplastic astrocytomas IDH-mutated (group 3), 4 anaplastic oligodendrogliomas and 2 low-grade oligodendrogliomas (together prognostic group 4). A mixed linear model evaluated the predictabilities of microvessel density (MVD), vascular area ratio (VAR), mean vessel size (MVS), vascular endothelial growth factor and receptors (VEGF-A, VEGFR‑2) and vascular endothelial-protein tyrosine phosphatase (VE-PTP) expression from Tofts model kinetic and model-free curve parameters.

**Results:**

All kinetic parameters were associated with VEGF‑A (all *p* < 0.001) expression. K^trans^, k_ep_ and v_e_ were associated with VAR (*p* = 0.006, 0.004 and 0.01, respectively) and MVS (*p* = 0.0001, 0.02 and 0.003, respectively) but not MVD (*p* = 0.84, 0.74 and 0.73, respectively). Prognostic groups differed in K^trans^ (*p* = 0.007) and v_e_ (*p* = 0.004) values measured in the infiltration zone. Despite significant differences of VAR, MVS, VEGF‑A, VEGFR‑2, and VE-PTP in vital tumor tissue and the infiltration zone (*p* = 0.0001 for all), there was no significant difference between kinetic parameters measured in these zones.

**Conclusion:**

The DCE-MRI kinetic parameters show correlations with microvascular parameters in vital tissue and also reveal blood-brain barrier abnormalities in the infiltration zones adequate to differentiate glioma prognostic groups.

**Supplementary Information:**

The online version of this article (10.1007/s00062-021-01015-3) contains supplementary material, which is available to authorized users.

## Introduction

Glioma vessels show multilevel abnormalities with hyperperfusion, a variable blood-brain barrier disruption (BBBD) and pathological vessel sizes and densities. Vascular endothelial growth factor (VEGF) driven angiogenesis is directly related to lesion malignancy and potential treatment susceptibility, lesion expansion, and secondary tumor dedifferentiation [1–3]. Monitoring microvessel status, including BBBD and the evolution of proangiogenic proteins, can be an essential feature to control the efficacy of glioma treatment [4].

Glioma vascularization can be assessed on a molecular level, e.g. through the analysis of expression of vascular endothelial growth factor (VEGF) the VEGF receptors (VEGFR) or vascular endothelial-protein tyrosine phosphatase (VE-PTP), which is associated with BBBD, as well as histologically studying microvessel density or presence of enlarged vessels (ESM 1) [5]. Gliomas are intraindividually heterogeneous [6, 7] and noninvasive methods are necessary to evaluate vascular proliferations to facilitate optimization of surgical resection planning. Magnetic resonance imaging (MRI) studies using dynamic susceptibility contrast MRI (DSC-MRI) delineated several vascular zones, or habitats, of distinct perfusion within a tumor. These have a prognostic relevance and extend beyond the glioma’s outer margins into the infiltration zone [8–10].

Dynamic contrast-enhanced MRI (DCE-MRI) is designed to provide a quantitative measure for BBBD [11, 12]. Its kinetic parameters contrast agent efflux transfer constant (K^trans^), contrast agent reflux transfer constant (k_ep_), the extracellular-extravascular volume fraction (v_e_) and the intravascular volume fraction (v_p_), a suspected correlate for vascular space, were shown to correlate with glioma WHO grades, genomic prognostic markers, microvascular anatomy such as vessel density, as well as VEGF expression [2, 3, 7, 13–18]. Kinetic parameters are influenced by both vessel perfusion and permeability and hence may be particularly useful to serve as imaging markers for vascular proliferation in gliomas (ESM 1). The reliability of DCE-MRI regarding the focal assessment of vascular parameters is, however, poorly examined [19]. This uncertainty includes the glioma infiltration zone, which only microscopically shows subtle tumor cell infiltration, especially in normal-appearing brain tissue [2, 20, 21]. Treatment planning and evaluation in patients with glioma must consider these peripheral regions. A meticulous confirmation of MRI kinetic parameters measurements is necessary to support the strength of MRI as a source of noninvasive biomarkers for vascular conditions in the brain. On the other hand, experimental DCE-MRI protocols showed enough sensitivity to detect very subtle BBBD in dementia patients [22]. The difficult aspect of a DCE-MRI study that involves analyses of lesions with a high permeability and perfusion next to lesions with variable or no permeability is that the kinetic models are not the same for all tissues. Not even the acquisition parameters of the dynamic sequence are optimal for all tissues. Highly perfused and permeable tumors will most likely be reliably assessed using the extended Tofts model, while lesions with low to no BBBD should be assessed with models sensitive to low BBBD, such as the Patlak model, or be analyzed model-free altogether [23]. There will thus be a trade-off in reliability or comparability, depending on whether one or several models are used to analyze these different tissue zones.

In order to advance the clinical implementation of DCE-MRI as a noninvasive method to evaluate glioma vascularization, this study aimed to explore if DCE-MRI kinetic parameters can focally predict histological and molecular vascular parameters in different vascular zones of and beyond glioma tissue and how this is related to the histology and expected prognosis of the lesion.

## Material and Methods

### Study Overview and Cohort Selection

This prospective study was approved by the university ethics committee (study approval number 358/13). Adult patients participated after written informed consent. Inclusion criteria were: 1) suspected or previously histologically confirmed glioma, 2) no contraindications against MRI, and 3) planned surgery. DCE-MRI was added to the standard preoperative MRI. Tissue samples covered vital tissue, the infiltration zone and visually healthy brain in areas needing to be resected anyhow. Tissue was diagnosed according to the 2016 WHO classification for central nervous system (CNS) tumors and analyzed for microvascular parameters, VEGF‑A, VEGFR‑2 and VE-PTP expression [24].

### MRI Acquisition and Processing

All patients were examined at 3.0 T (Achieva TX, Philips Healthcare, Best, The Netherlands) using an 8‑channel head coil. The 3D contrast-enhanced T1-weighted sequence was acquired for intraoperative neuronavigation (TR 7.5 msec, TE 3.5 msec; FA 8 °, FOV 256 × 256 × 256 mm, slices 170, scan time 4 min 40 sec, voxel size 1.0 × 1.0 × 1.0 mm^3^). The DCE-MRI sequence involved a double flip-angle technique and 50 dynamic scans (whole-brain coverage, FOV 108 × 220 × 182mm, 36 slices, voxel size 1.57 × 1.6 × 3 mm, scan time 5 min 51 sec; T1 reference scans: TR 10 msec, TE 2.3 msec, flip angles 5/15°; dynamic scans (5.3 sec each): TR 3.5 msec, TE 2.3 msec, flip angle 8°). Gadobutrol (Gadovist®, Bayer Healthcare, Leverkusen, Germany; standard dose 0.1 mmol/kg body weight) was the contrast agent injected at 3 mL/s through an antecubital vein followed by 25 mL flush saline. The assumed relaxivity at 37 °C body temperature was 5.0/(mmol*s). Kinetic parameters were calculated based on the extended Tofts model with an arterial input function derived from individually placed regions of interest (ROI) in the superior sagittal sinus (Intellispace Portal 5.0, Philips Healthcare, Best, The Netherlands). Choosing the extended Tofts model for all ROI including healthy brain is a compromise to maintain comparability of the data. An additional model-free analysis of the area under the receiver operating characteristic curve (AUC) and the maximum relative enhancement (mrE) were an approach to compensate for this circumstance. The mrE is defined as the maximum signal difference compared to baseline in percent (%).

**Fig. 1:**
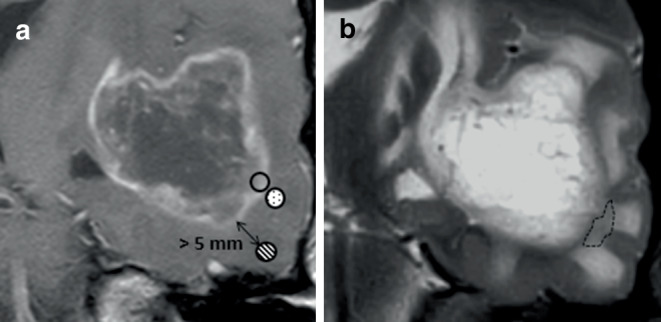
Tissue zones in glioblastoma. **a** Partial coronal view of a left temporal glioblastoma in a 56-year-old man (T1-weighted plus contrast). The *clear black circle* marks vital glioma tissue (zone 1). The *adjacent dotted circle* marks the infiltration zone (zone 2). The *striped circle* defined the suspected zone 3 (visually healthy tissue) in a minimum distance of 5 mm to the enhancing glioma rim. **b** The corresponding T2-weighted image serves as a radiological verification of glioma zones with zone 2 being in a T2 hypointense area (*striped outline*), while zone 3 represents normal cortex and subcortical mild vasogenic edema. N.B. tissue had to appear healthy intraoperatively under the microscope

Kinetic parameter maps were fused with the 3D neuronavigation datasets to be used for ROI placement. To measure kinetic parameters, a neuroradiologist (V.C.K.) selected 0.5 × 0.5 × 0.5 cm^3^ ROI in the following zones (Fig. 1):

- Zone 1: contrast-enhancing glioma and/or T2-hyperintense tissue,
- Zone 2: suspected infiltration zone,
- Zone 3: visually healthy brain tissue with more than 5 mm distance to the outer contrast-enhancing area of the tumor with either high signal on T2 or brain-isointense signal.

The total number of planned biopsies varied by tumor size and safe resectability of peripheral tissue in zones 2 and 3 from 2–6 biopsies per patient.

### Tissue Preparation and Analyses

Kinetic parameter corresponding biopsies were identified by pointer navigation in Softtouch® referencing technique (M.S. and B.P., Brainlab AG, Munich, Germany) based on the marked ROI and were all successfully retrieved at the beginning of surgery to minimize brain shift effects. They were formalin-fixed and paraffin-embedded. Routine neuropathological diagnostics to achieve a diagnosis according to the revised 4th edition of the 2016 WHO classification of tumors of the CNS was performed on independently taken biopsy tissue (G.H.G.) [24]. Based on the publication by Hartmann et al. four prognostic groups were created out of all patients’ WHO 2016 diagnoses: 1) short survival: glioblastoma °IV and astrocytoma °III, both IDH-wildtype; 2) shorter intermediate survival: glioblastoma °IV, IDH-mutated; 3) longer intermediate survival: astrocytoma °III, IDH-mutated and 4) longer survival: oligodendroglioma °III and °II, IDH-mutated, 1p/19q co-deleted [25]. In prognostic group 1, zone 1 biopsies, which—different from preoperative expectation on MRI—histologically identified as mainly necrotic were only analyzed concerning descriptive statistics of the respective microvascular and kinetic parameters, but were not considered in prognostic group or zone analyses.

For microvessel analysis, 4 µm thick tissue specimens were stained against endothelial marker *CD34* (Ventana Benchmark XT Immunostainer, Roche Ventana, Darmstadt, Germany; 1/100 antibody: DAKO, Glostrup, Denmark). Digital scans of the tissue slides were analyzed with quantification software (Tissue Studio®, Definiens, Munich, Germany). Automated settings were subjected to manual training adjustments in order to exclude artefacts (K.H.). Microvessels were defined as vessel areas smaller than 2.0 mm^2^. The software defined microvascular parameters based on 0.6 × 0.6 mm^2^ target squares and t3 microvascular parameters (vascular area ratio, VAR, microvessel density, MVD, mean microvessel size, MVS) were determined. To calculate the VAR, which is the percentage of area covered by blood vessels in comparison to the area covered by other tissues on the slide, all single areas of vascular tissue (including their lumina) were added and divided by the remaining nonvascular tissue area. Vascularization data of all analyzed tiles in a biopsy section were averaged to the mean.

For VE-PTP, 4 µm thick tissue sections were stained with purified mouse anti-human VEGF‑A (clone G153-694) and VE-PTP (BD Pharmingen, Heidelberg, Germany; clone 12; indirect immunohistochemistry). Antigen retrieval was achieved using 90 °C target retrieval solution (DAKO, Glostrup, Denmark) and washing in phosphate buffered saline with 0.3% Triton X‑100 (Sigma Aldrich, Steinheim, Germany). Primary antibody (VE-PTP 1:150) was diluted in 1% bovine serum antigen and stained at 4 °C. Secondary 1/500 biotinylated goat anti-mouse-antibody (DAKO, Glostrup, Denmark) was applied for 45 min at room temperature. Amplification involved the ABC-(HRP)-kit (VECTA Laboratories, Peterborough, UK) and 3,3′-diaminobenzidine was the chromogenic substrate (VECTA Laboratories, Peterborough, UK). IgG-negative controls were run. Free VE-PTP expression was assessed by the scoring system of Raica et al. [26]. Intensity and percentage of VE-PTP positive stained cells were analyzed.

Tumor sections (4 µm) were subjected to immunohistochemistry for VEGF‑A and VEGFR‑2 using mouse IgG2b anti-human VEGF (clone: MAB293, dilution 1:100; R&D Systems, Minneapolis, MN, USA) and monoclonal rabbit anti-human VEGFR‑2 dilution 1:500 (clone: 55B11; Cell Signaling, Danvers, MA, USA). Tissue labeling was performed using the Leica Bond III immunohistochemistry system (Leica Biosystems, Buffalo Grove, IL, USA). For VEGF‑A, a cell conditioning pretreatment with enzyme was performed for 10 min, for VEGFR‑2 cell conditioning was performed with EDTA for 20 min. The primary antibodies were applied for 15 min. Standard secondary antibody (Leica Biosystems) was incubated for 8 min. For diaminobenzidine (DAB) visualization and counterstaining, the Bond Polymer Refine Detection Kit (Leica Biosystems) was used. High overlap with mRNA levels demonstrated by in situ hybridization for the soluble VEGF‑A could be proven [27, 28].

For quantification of VEGF‑A and VEGFR‑2 expression, a semiquantitative score was used. The immunohistochemical staining intensity (negative = 0, low = 1, moderate = 2, strong = 3) was multiplied by the proportion of positive tumor or vascular cells separately. Each product was then added to each other receiving a sum score. This histological score is well evaluated as published before [29, 30].

### Statistics

Statistical analyses were performed with SPSS 24.0 (IBM, Armonk, NY, USA). First, K^trans^ kinetic parameter results were Pearson correlated with AUC and mrE of the signal intensity time curves to explore if model-free measurements, which are possibly less affected by false results due to low permeability than kinetic parameters of the Tofts model, are related to kinetic parameters. This was taken as an indirect corroboration of the reliability of the kinetic parameters. The operation was performed for all tissue zones separately. The DCE-MRI kinetic parameters were explored regarding their capacity to predict microvascular parameters, VEGF‑A, VEGFR‑2 and VE-PTP scores in a generalized linear (dichotomous data) or linear mixed (continuous data) model approach with the patient as the random factor. A linear mixed model similarly determined tissue zone-dependent differences of parameters. Kruskal-Wallis tests were used to identify parameter differences between prognostic groups measured in the same zones. The ROC analyses determined the discriminability tissue zones or prognostic groups by kinetic parameters. Analyses were performed with inclusion of recurrent cases after evaluation of significant parameter differences (Whitney-Mann U‑test stratified by tumor zones and prognostic groups) in these groups to rule out possible effects of chemoradiotherapy. Multiple testing correction for linear mixed models is difficult. All other results are also stated after Bonferroni correction.

## Results

### Cohort Demographics, Biopsy Overview and Parameter Correlations

Patients and diagnoses as well as biopsy numbers are listed in ESM 2. An overview of the biopsies, the histology and tissue zones, is presented in Tables 1 and 2. An example of tissue zones in one individual is given in ESM 3. Note that tissue presumed to lie in zone 2 or 3 indeed was later rated as infiltration zone or normal brain in all cases, while 14 out of 68 planned biopsies in vital tumor turned out to be mainly necrotic tissue reducing the amount of analyzed zone 1 to 3 biopsies to 106 (all in IDH-wildtype GBM, Tables 1 and 2). The mean kinetic parameter values and model-free values are presented in ESM 4.

**Table 1:** Biopsies and diagnoses

**WHO 2016 diagnosis including tumor grades**			
**–**	**Number of patients and biopsies**	**Prognostic groups according to WHO 2016**	**Number of patients and biopsies**
GBM °IV IDHwt	*n* = 17 (71 biopsies)	*(1) short survival:* GBM °IV and Astrocytoma °III, IDHwt	*n* = 18 (75 biopsies)
Astrocytoma °III, IDHwt	*n* =1 (4 biopsies)		
GBM °IV, IDHmut	*n* =3 (15 biopsies)	*(2) shorter intermediate survival:* GBM °IV, IDHmut	*n* =3 (15 biopsies)
Astrocytoma °III, IDHmut	*n* =3 (8 biopsies)	*(3) longer intermediate survival:* Astrocytoma °III, IDHmut	*n* =3 (8 biopsies)
ODG °III, IDHmut	*n* =4 (16 biopsies)	*(4) longer survival:* ODG °III and °II, IDHmut	*n* =6 (22 biopsies)
ODG °II, IDHmut	*n* =2 (6 biopsies)		
**Number of biopsies by WHO 2016 grades**			
°IV	86	–	
°III	28	–	
°II	6	–	

*n* = 30 patients (9 women, 38 biopsies; 21 men, 82 biopsies)

Total biopsies *n* = 120 (*n* = 94 de novo tumors, *n* = 26 recurrent cases)

*GBM* glioblastoma, *IDHwt/mut* isocitrate dehydrogenase wildtype/mutated (or mutant), *ODG* oligodendroglioma, *WHO* World Health Organization

**Table 2:** Biopsy zone distribution by histopathological confirmation

	Total	By WHO 2016 prognostic group			
		1	2	3	4
Necrosis	14 biopsies^a^	14^a^	–	–	–
Vital tumor (zone 1)	54 biopsies	35	8	4	7
Infiltration zone (zone 2)	33 biopsies	15	4	3	11
Normal brain (zone 3)	19 biopsies	11	3	1	4

*n* = 30 patients (9 women, 38 biopsies; 21 men, 82 biopsies)

Total biopsies *n* = 120 (*n* = 94 de novo tumors, *n* = 26 recurrent cases)

*WHO* World Health Organization

^a^ not used in further comparative zone analyses being a necrotic biopsy

The correlation coefficient for K^trans^ with the AUC was 0.78 (*p* < 0.0001) and 0.85 (*p* < 0.0001; *n* = 106 biopsies/ROI) with the mrE when considering all biopsies. Taking only zones 2 and 3 ROI into account these values were 0.92 and 0.90, respectively (*n* = 52).

### Relationship of Kinetic MRI Parameters with Vascular Parameters

All cases (*n* = 30, 106 biopsies) were considered in this analysis due to a lack of significant parameter value differences between de novo and recurrent cases. The K^trans^, k_ep_ and v_e_ were significantly associated with VAR (*p* = 0.006, 0.004 and 0.01, respectively) and MVS (*p* = 0.0001, 0.02 and 0.003, respectively), but not MVD (*p* = 0.84, 0.74 and 0.73, respectively). The v_p_ was only significantly associated with MVS (*p* = 0.002), but not VAR (*p* = 0.09) or MVD (*p* = 0.93).

Looking at biopsies including all tissue zones (*n* = 106 biopsies), all kinetic parameters were significantly associated with VEGF‑A expression (all *p* < 0.001), while VEGFR‑2 expression did not reach a significant association with K^trans^, v_e_ or v_p_ (*p* = 0.08, 0.13 and 0.15 respectively), but with k_ep_ (*p* = 0.04) only.

There was no association between any of the kinetic parameters with VE-PTP expression (K^trans^ *p* = 0.13, k_ep_ *p* = 0.12, v_e_ *p* = 0.44 and v_p_ *p* = 0.27).

### Relevance and Identification of Tissue Zones Regarding Kinetic and Microvascular Parameters

**Fig. 2:**
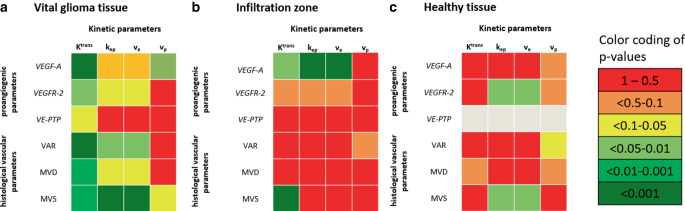
Heat map presentation of correlations between kinetic and vascular parameters.** a** Vital glioma tissue zone 1, **b** infiltration zone 2, **c** healthy appearing tissue zone 3. N.B. no statistics could be performed for VE-PTP in healthy appearing tissue as all samples showed the same nonexpression (score 0, expressed *in grey*)

The detailed analysis split by single tissue zones (compare Tables 1 and 2) showed that the association between kinetic parameters and vascular parameters was largely based on the vital glioma tissue, and that K^trans^ was the most reliably correlated kinetic parameter (Fig. 2); however, VEGF‑A showed an association with more kinetic parameters in the infiltration zone rather than in vital tumor tissue (Fig. 2).

Regarding the differentiability of vital tumor from the infiltration zone in gliomas of the same prognostic group (compare Tables 1 and 2), VAR (2.9 ± 0.4 vital tumor vs. 1.1 ± 0.1 infiltration zone vs. 1.3 ± 0.1 normal brain, in %), MVS (387.1 ± 27.5 vital tumor vs. 193.5 ± 15.6 infiltration zone vs. 178.3 ± 17.5 normal brain) as well as VEGF‑A (42.3 ± 9.8 vital tumor vs. 9.5 ± 7.2 infiltration zone vs. 0.3 ± 0.3 normal brain), VEGFR‑2 (108.7 ± 14.3 vital tumor vs. 18.3 ± 9.3 infiltration zone vs. 4.3 ± 2.0 normal brain) and VE-PTP (0.5 ± 0.1 vital tumor vs. 0.1 ± 0.1 infiltration zone vs. 0 normal brain) scores were all significantly higher in vital tumor tissue than in the infiltration zones or normal brain (*p* = 0.0001 for all; mixed linear model); however, kinetic parameters did not differ significantly between zones (ESM 4).

**Fig. 3:**
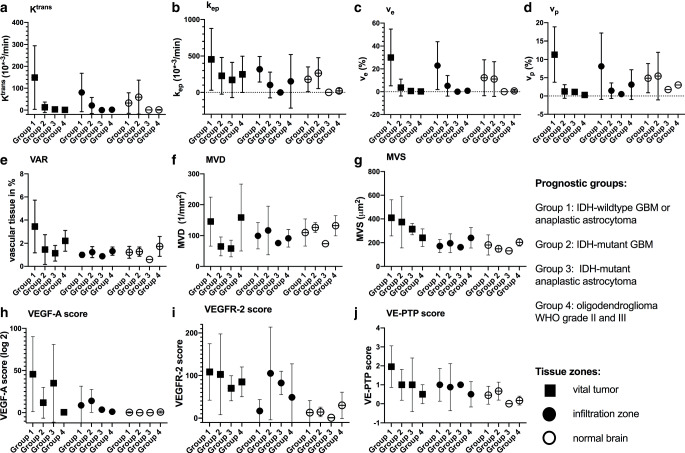
Differences of kinetic and vascular parameters between tissue zones and prognostic groups. **a**–**d** Kinetic parameters, **e**–**g** histological microvascular parameters, **h**–**k** vascular growth factors (*group* *1* short survival, glioblastoma °IV, IDH-wildtype, *group* *2* shorter intermediate survival, glioblastoma °IV, IDH-mutant, *group* *3* longer intermediate survival, astrocytoma °III, IDH-mutated, *group* *4* longer survival, oligodendroglioma °III and °II, IDH-mutant, 1p/19q co-deleted, *K*^*trans*^ contrast agent transfer constant (efflux to EES), *k*_*ep*_ contrast agent transfer constant (reflux to vessels), *v*_*e*_ EES volume fraction (proposed cellularity marker), *v*_*p*_ plasma volume fraction (proposed marker for vascularization), *VAR* vascular area ratio, *MVD* mean vessel density, *MVS* mean vessel size, *VEGF‑A* vascular endothelial growth factor type A, *VEGFR‑2* vascular endothelial growth factor receptor type 2, *VE-PTP* vascular endothelial-protein tyrosine phosphatase)

Fig. 3 presents the detailed tissue zones and prognostic group dependent parameter value distribution.

### Prognostic Tumor Groups Regarding Kinetic and Microvascular Parameters and Different Tissue Zones

**Fig. 4:**
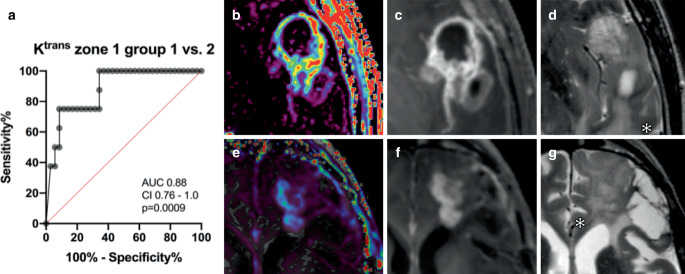
Example of the prognostic group differentiation potential of K^trans^ measured in vital tumor of groups 1 and 2.** a** ROC curve, **b**–**d** example of a 56-year-old patient in prognostic group 1 with a GBM, IDH-wildtype, **e**–**g** example of a 77-year-old patient in prognostic group 2 with a GBM, IDH-mutant, **b**, **e** K^trans^ map, **c**, **f** contrast-enhanced T1-weighted, **d**, **g** T2 TSE. All images are axial. Asterisks in (**d**) and (**g**) mark areas defined as infiltration zone. N.B. these two groups differ in their IDH status and therefore this graph also depicts the IDH status differentiation potential of K^trans^ measured in vital high-grade glioma tissue

Comparing vital tumor (zone 1) between the four prognostic tumor groups (compare Tables 1 and 2) K^trans^ (*p* = 0.001), v_e_ (*p* = 0.002) and v_p_ (*p* = 0.0001) differed significantly, but not k_ep_ (*p* = 0.337). The K^trans^ measured in vital tumor zone 1 was a suitable parameter to differentiate prognostic group 1 from group 2 (AUC = 0.88, CI 0.76–1.0, *p* = 0.0009; Fig. 4). The VAR (*p* = 0.008) and the MVD (*p* = 0.009) in zone 1 also differed between prognostic groups. This significance was, however, based on the large difference of one prognostic group differing more than the others (Fig. 3). Comparing measurements in zone 1, groups 2–4 did not significantly differ between each other in any of the parameters including VEGF‑A, VEGFR‑2 and VE-PTP (Fig. 3 and ESM 4 for kinetic parameters).

The K^trans^ (*p* = 0.007), k_ep_ (*p* = 0.019) and v_e_ (*p* = 0.004) measured in the infiltration zone were significantly different between prognostic groups due to higher kinetic parameter values again in prognostic group 1 (ESM 4). In the infiltration zone, prognostic groups 1 and 2 could be differentiated to some degree (AUC = 0.81, CI 0.52–1.0, *p* = 0.05) using K^trans^. No differences in kinetic parameters were noted when comparing between prognostic groups in normal brain despite higher mean K^trans^ and k_ep_ in prognostic groups 1 and 2 than in groups 3 and 4.

## Discussion

This interdisciplinary glioma study suggests that vascular growth factors, microvascular anatomy, and DCE kinetic parameters show significant correlations not only in vital tumor tissue, but also in the histologically confirmed infiltration zone of gliomas, albeit to a lesser degree. Second, kinetic parameters and histological microvascular parameters also differed between the group of gliomas with the worst prognosis (glioblastoma, IDH-wildtype) and all other groups when comparing values measured in the infiltration zones of this group versus the others. Finally, VEGF‑A showed a broader association with kinetic parameters in tissue of the infiltration zone rather than in the vital tumor tissue.

Substantial percentages of high-grade gliomas do not show avid contrast enhancement [31]. The exchange of contrast agent between the extravascular and intravascular spaces is a multifactorial process influenced by the BBB, quantitative and qualitative aspects of the microvascular network and cerebral blood flow (i.e., perfusion; ESM 1) [32]. One of our hypotheses was that BBBD could be estimated directly by the presence of free vascular endothelial-protein tyrosine phosphatase (VE-PTP), which dissociates from tight junction re-enforcing protein VE-cadherin. Consequently, tight junctions between endothelial cells open and vessels become permeable [5], which would be reflected by DCE-MRI kinetic parameters; however, we could not identify any association between VE-PTP expression and any of the kinetic parameters. While anti-VEGF drug effects seem to be reliably observable using DCE-MRI [3], this is already less established for proangiogenic factors, such as VEGF‑A expression itself. In a study by Jensen et al., and confirmed by our results, v_p_ was the only kinetic parameter that correlated to a significant degree with VEGF‑A [2]. In contrast, in the study of Awasthi et al. VEGF‑A expression was correlated exclusively with k_ep_ [13] and in the study by Di et al. exclusively with K^trans^ [33], showing the low degree of data coherence. Di et al. could further only establish an association in vital tumor areas of high-grade glioma.

A strength of the present study is that multiple biopsies were taken in regions outside the apparent tumor and even in healthy tissue. Kinetic parameters mostly reflected microvascular parameters poorly outside the vital tumor. Limited detectability of BBBD with standard DCE-MRI is also a known obstacle in other areas such as dementia imaging [34]. As an exception, VEGF‑A was associated more broadly with kinetic parameters in the infiltration zone than in vital tumor. The MVS was the only microvascular parameter showing association with kinetic parameters (here K^trans^) in the infiltration zone. These findings corroborate those of Tamura et al. regarding the unique features of VEGF expression and microvascularization of the infiltration zone in glioma [35].

Kinetic parameters did not differ between the vital tissue and infiltration zone of glioma of the same prognostic group but differed significantly between prognostic groups when comparing measurements in the same zones. As the IDH mutation status differed between prognostic groups 1 and 2, kinetic parameters could differentiate these when measured in both vital tumor and also infiltration zones, which is contradictory to findings by others for DCE-MRI [16, 18]. It is noteworthy that despite not reaching a significant level of difference K^trans^ measured in healthy-rated tissue of prognostic groups 1 and 2 was minimally elevated compared to groups 3 and 4, which showed the expected virtual zero kinetic parameter values. This is an unexpected finding; however, the microscopically normally appearing biopsies came from regions, which were, after all, still very close to the tumor and not from distant locations. It might be possible that the BBB is already compromised at this point. The prognostically different groups 3 and 4 did not differ in radiological parameters. All of these findings imply that DCE-MRI has some potential to be used as a noninvasive advanced MRI method to assess gliomas beyond the vital tumor center, e.g., regarding the necessity for resection beyond the contrast-enhancing tumor boundaries. Furthermore, it stresses currently unsolved, but relevant questions regarding the reliability of kinetic parameter measurements in healthy brain tissue of glioma patients. The DCE-MRI sequences and post-processing adapted to low leakage may be a key solution.

However, in vital tumor tissue, kinetic parameters in this study also correlated with VAR and MVS, and more reliably than MVD. This finding corroborates the idea that large vessels and total vascularity of the examined tissue contribute more to the measurement than the sheer density of many small vessels. A similar finding was expressed very recently in a breast cancer model [36] and by Di et al. [19]. MVD differing significantly between prognostic groups supports that differences exist, but that DCE-MRI cannot show them. Notably, the parameter v_p_ was not associated with any of the microvascular histological parameters in this study, despite being considered a substitute marker for vascularity and its high repeatability found even in healthy brain tissue [37]. Reasons for a limited comparability between results are, however, abundant in studies with quantitative MRI techniques. In the case of a v_p_ analysis the arterial input function selection, e.g., can be an influential factor on results. At this point the choice of kinetic model needs to be addressed. While the extended Tofts model is generally not recommended for lesion analyses in low permeability regions, this compromise needed to be made for reasons of comparability of analyses between the tissue zones and histologically different lesions. The high correlations of presumably more robust model-free parameters, AUC and mrE, with the kinetic parameter K^trans^ also in infiltration zone and adjacent normal brain can be interpreted as an indicator that kinetic parameter measurements in these peripheral zones are still acceptably correct, albeit not ideal. This is supported by the work of Cramer et al., who showed a greater reliability for the Patlak model but a limited robustness also for the Tofts model, too, in low permeability tissues [23].

The large MR morphological differences in all parameters within corresponding regions and prognostic groups indicate a high variability of vascularization and VEGF‑A expression. They also stress the problem of interindividual differences in kinetic parameters, also seen in this study, which hamper the establishment of cut-off values for lesion differentiation, and demanded a statistical approach balancing these differences in this study. It is possibly surprising that biopsies from contrast-enhancing regions of glioblastoma were evaluated as necrotic rather than vital tissue despite comparable kinetic values and careful handling of the tissue. An explanation could be a close proximity to necrosis in a region sometimes styled as the hypoxic penumbra of centrally necrotic glioma, which is an enhancing transition region to central necrosis [2].

### Limitations

The numbers of patients and biopsies, especially in lower grade tumors, are limited and unevenly distributed. Only limited amounts of normal appearing brain tissue could be retrieved to avoid higher operative risk for patients. Another aspect is the clear separation of the different tumor areas and zones, which was based on classical neuroradiological criteria for this study; however, it could be assessed by (additive or alternative) artificial intelligence-based methods for future studies as are already available [38].

## Conclusion

DCE-MRI seems sensitive for changes in the blood-brain barrier beyond the vital glioma tissue itself and may differentiate prognostically different gliomas in the infiltration zone and show an association with VEGF‑A expression in this region.

## Supplementary Information

ESM 1: Assumed interaction of blood-brain barrier, vascular environment and DCE-MRI kinetic parameters in glioma. Glioma cells express VEGF, which triggers the dissociation of VE-PTP from VE-cadherin and thereby induces an elevated vascular permeability in tumor microvessels. VEGF also induces neovascularization. As a result, vessel density and leakage of BBB assumingly rise with tumor grade. This is possibly associated with kinetic parameters of “permeability MRI”, DCE-MRI. DCE-MRI parameters, especially K^trans^, are modulated by perfusion (cerebral blood flow, CBF) and the permeable vessel surface area product (PS). The relationship between kinetic and microvascular parameters and BBB disruption inducing proteins VEGF and VE-PTP is unclear. *BBB* blood-brain barrier, *CBF* cerebral blood flow, *EES* extravascular-extracellular space, *GdCA* gadolinium-based contrast agent, *K*^*trans*^ contrast agent transfer constant (efflux to EES), *k*_*ep*_ contrast agent transfer constant (reflux to vessels), *PS* permeability-surface area product, *VE-PTP* vascular endothelial-protein tyrosine phosphatase, *VE-cadherin* vascular endothelial cadherin, *VEGFR‑2* vascular endothelial growth factor receptor 2, *v*_*e*_ EES volume fraction (proposed cellularity marker), *v*_*p*_ plasma volume fraction (proposed marker for vascularization).

ESM 2: Patient cohort overview

ESM 3: Microscopic impression of tumor zones in tissue of a 77-year-old patient with an IDH-wildtype glioblastoma.** a** Vital tumor zone 1 marked with already size-categorized vessels during post-processing, **b** infiltration zone 2 showing also basic vessel registration in purple (walls) and green (lumen), **c** tissue sample taken from the inner contrast enhancing rim and turning out as mainly necrotic, **d** zone 3 normal appearing tissue showing no cellular atypia, vessel proliferations or necrosis.

ESM 4: kinetic parameter values by tissue zone and prognostic group
