# Supplementary figures and images for: DCE-MRI in Glioma, Infiltration Zone and Healthy Brain to Assess Angiogenesis: A Biopsy Study
Source: Clin Neuroradiol. 2021 Apr 26;31(4):1049–58. doi: 10.1007/s00062-021-01015-3 (PMC8648693; doi:10.1007/s00062-021-01015-3)

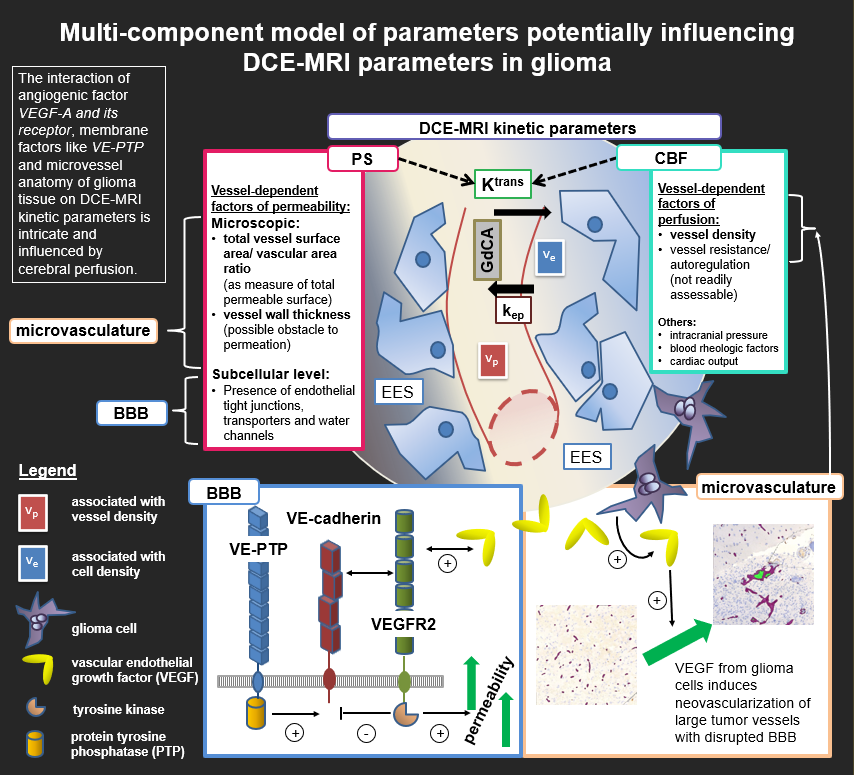

Supplement: Supplementary file 1 — ESM 1: Assumed interaction of blood-brain barrier, vascular environment and DCE-MRI kinetic parameters in glioma. Glioma cells express VEGF, which triggers the dissociation of VE-PTP from VE-cadherin and thereby induces an elevated vascular permeability in tumor microvessels. VEGF also induces neovascularization. As a result, vessel density and leakage of BBB assumingly rise with tumor grade. This is possibly associated with kinetic parameters of “permeability MRI”, DCE-MRI. DCE-MRI parameters, especially Ktrans, are modulated by perfusion (cerebral blood flow, CBF) and the permeable vessel surface area product (PS). The relationship between kinetic and microvascular parameters and BBB disruption inducing proteins VEGF and VE-PTP is unclear. BBB blood-brain barrier, CBF cerebral blood flow, EES extravascular-extracellular space, GdCA gadolinium-based contrast agent, Ktrans contrast agent transfer constant (efflux to EES), kep contrast agent transfer constant (reflux to vessels), PS permeability-surface area product, VE-PTP vascular endothelial-protein tyrosine phosphatase, VE-cadherin vascular endothelial cadherin, VEGFR‑2 vascular endothelial growth factor receptor 2, ve EES volume fraction (proposed cellularity marker), vp plasma volume fraction (proposed marker for vascularization). [file 62_2021_1015_MOESM1_ESM.tiff]

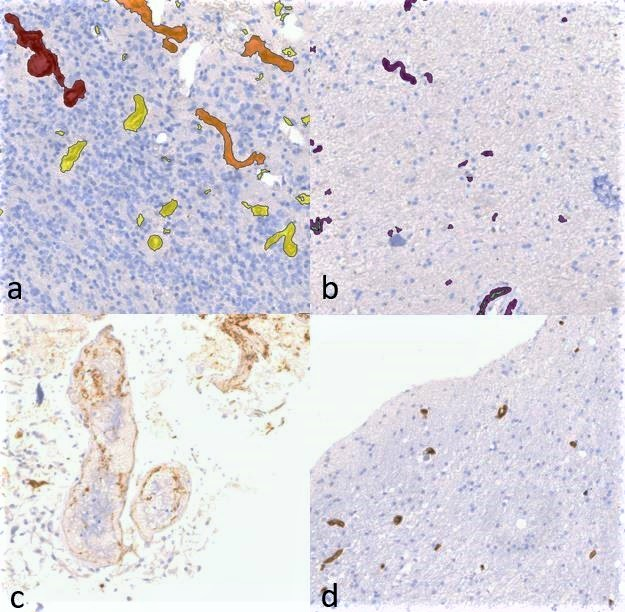

Supplement: Supplementary file 3 — ESM 3: Microscopic impression of tumor zones in tissue of a 77-year-old patient with an IDH-wildtype glioblastoma. a Vital tumor zone 1 marked with already size-categorized vessels during post-processing, b infiltration zone 2 showing also basic vessel registration in purple (walls) and green (lumen), c tissue sample taken from the inner contrast enhancing rim and turning out as mainly necrotic, d zone 3 normal appearing tissue showing no cellular atypia, vessel proliferations or necrosis. [file 62_2021_1015_MOESM3_ESM.tiff]
