# Supplementary material for: DCE-MRI in Glioma, Infiltration Zone and Healthy Brain to Assess Angiogenesis: A Biopsy Study
Source: Clin Neuroradiol. 2021 Apr 26;31(4):1049–58. doi: 10.1007/s00062-021-01015-3 (PMC8648693; doi:10.1007/s00062-021-01015-3)
Supplement: Supplementary file 2 — ESM 2: Patient cohort overview [file 62_2021_1015_MOESM2_ESM.docx]

| **ESM 1 Patient cohort overview** | | | | | | | | | | | |
| --- | --- | --- | --- | --- | --- | --- | --- | --- | --- | --- | --- |
| Patient | Sex | Age | OS | Entity | Number of biopsies | Visible CE | IDH status | 1p/19q  co-deletion | WHO grade | recurrent | WHO 2007 diagnosis |
| 1 | 1 | 63 | 9 | GBM | 4 | 1 | wt | n/a | °IV | 0 |  |
| 2 | 1 | 47 | 20 | GBM | 5 | 1 | wt | n/a | °IV | 0 |  |
| 3 | 0 | 47 | 20 | GBM | 3 | 1 | wt | n/a | °IV | 0 |  |
| 4 | 0 | 41 | 7 | GBM | 3 | 1 | wt | n/a | °IV | 1 | GBM °IV |
| 5 | 0 | 77 | 7 | GBM | 5 | 1 | wt | n/a | °IV | 0 |  |
| 6 | 1 | 69 | 14 | GBM | 4 | 1 | wt | n/a | °IV | 0 |  |
| 7 | 1 | 76 | 12 | GBM | 3 | 1 | wt | n/a | °IV | 0 |  |
| 8 | 0 | 66 | 35 | GBM | 6 | 1 | wt | n/a | °IV | 0 |  |
| 9 | 0 | 56 | 38 | GBM | 4 | 1 | wt | n/a | °IV | 0 |  |
| 10 | 0 | 56 | 37 | GBM | 4 | 1 | wt | n/a | °IV | 0 |  |
| 11 | 1 | 65 | 14 | GBM | 5 | 1 | wt | n/a | °IV | 0 |  |
| 12 | 0 | 79 | 4 | GBM | 5 | 1 | wt | n/a | °IV | 0 |  |
| 13 | 1 | 72 | 32 | GBM | 4 | 1 | wt | n/a | °IV | 0 |  |
| 14 | 1 | 69 | 4 | GBM | 5 | 1 | wt | n/a | °IV | 0 |  |
| 15 | 1 | 56 | 32 | GBM | 4 | 1 | wt | 0 | °IV | 0 |  |
| 16 | 1 | 77 | 5 | GBM | 5 | 1 | mut | n/a | °IV | 1 | GBM °IV |
| 17 | 1 | 46 | 32 | GBM | 4 | 0 | mut | n/a | °IV | 0 |  |
| 18 | 1 | 45 | 6 | GBM | 6 | 0 | mut | 0 | °IV | 1 | OA °II |
| 19 | 1 | 52 | 10 | Giant cell GBM | 4 | 1 | wt | n/a | °IV | 0 |  |
| 20 | 1 | 60 | 19 | Gliosarcoma | 3 | 1 | wt | n/a | °IV | 0 |  |
| 21 | 1 | 42 | 38 | Astrocytoma | 4 | 1 | wt | n/a | °III | 0 |  |
| 22 | 1 | 39 | 38 | Astrocytoma | 3 | 0 | mut | 0 | °III | 1 | Astrocytoma °III |
| 23 | 0 | 39 | 24 | Astrocytoma | 3 | 1 | mut | 0 | °III | 0 |  |
| 24 | 1 | 37 | 35 | Astrocytoma | 2 | 1 | mut | 0 | °III | 1 | OA °III |
| 25 | 1 | 56 | 7 | ODG | 4 | 0 | mut | 1 | °III | 1 | ODG °II |
| 26 | 1 | 58 | 32 | ODG | 4 | 0 | mut | 1 | °III | 0 |  |
| 27 | 1 | 55 | 41 | ODG | 3 | 0 | mut | 1 | °III | 1 | OA °II |
| 28 | 0 | 30 | 35 | ODG | 5 | 0 | mut | 1 | °III | 0 |  |
| 29 | 1 | 39 | 32 | ODG | 4 | 0 | mut | 1 | °II | 0 |  |
| 30 | 1 | 43 | 64 | ODG | 2 | 0 | mut | 1 | °II | 0 |  |
| **Caption:** Overview of the patient cohort demographics. N.B.: Recurrent cases and their diagnoses were initially diagnosed according to WHO 2007 classification and re-classified according to the 2016 WHO classification. CE: contrast enhancement; GBM: glioblastoma; IDH: isocitrate dehydrogenase; mut: mutant/mutated; OS: overall survival; OA: oligoastrocytoma; ODG: oligodendroglioma; wt: wildtype; 0: no/female; 1: yes/male | | | | | | | | | | | |
