# Supplementary material for: DCE-MRI in Glioma, Infiltration Zone and Healthy Brain to Assess Angiogenesis: A Biopsy Study
Source: Clin Neuroradiol. 2021 Apr 26;31(4):1049–58. doi: 10.1007/s00062-021-01015-3 (PMC8648693; doi:10.1007/s00062-021-01015-3)
Supplement: Supplementary file 4 — ESM 4: kinetic parameter values by tissue zone and prognostic group [file 62_2021_1015_MOESM4_ESM.docx]

| **ESM 4 kinetic parameter values by tissue zone and prognostic group** | | | | | |
| --- | --- | --- | --- | --- | --- |
| **Prognostic group** | **Kinetic parameter** | **Necrosis** | **Zone 1** | **Zone 2** | **Zone 3** |
|  |  | **(mean ± standard deviation, 95% confidence interval)** | | | |
| **Group 1** | **K^trans^**  **k_ep_**  **v_e_**  **v_p_**  **AUC**  **mrE** | 139.6±109.7  (82.2 – 197.1)  409.0±205.8  (301.3 – 516.8)  28.7±17.9  (19.3 – 38.1)  10.6±7.7  (6.6 – 14.6)  7.6±4.1  (5.2 – 9.9)  99.4±58.5  (65.6 – 133.2) | 136.2±133.4  (91.0 – 181.3)  434.7±377.4  (307.0 – 562.4)  28.5±22.8  (20.8 – 36.3)  10.7±7.7  (8.1 – 13.3)  8.2±6.7  (2.6 – 13.8)  69.4±53.4  (24.8 – 114.0) | 68.6±85.2  (21.4 – 115.7)  298.8±179.2  (199.6 – 398.0)  19.2±20.7  (7.7 – 30.7)  6.6±8.6  (1.8 – 11.4)  7.4±7.0  (2.5 – 12.5)  71.9±77.2  (16.7 – 127.2) | 32.5 **±**49.1  (0.0 – 65.5)  180.4**±** 178.9  (60.3 – 300.6)  12.2 **±**16.5  (1.1 – 23.3)  4.9 **±**4.2  (2.1 – 7.7)  3.5±4.9  (0 – 7.2)  33.9±57.41  (0 – 78.1) |
| **Group 2** | **K^trans^**  **k_ep_**  **v_e_**  **v_p_**  **AUC**  **mrE** | - | 15.6±26.0  (0.0 – 37.3)  286.1±259.6  (69.1 – 503.1)  3.8±7.3  (0.0 – 9.9)  1.3±1.9  (0.0 – 2.9)  1.65±1.7  (0 – 14.0)  21.61±32.5  (0 – 55.7) | 21.2±42.5  (0.0 – 88.8)  102.2±204.3  (0.0 – 427.3)  5.2±10.4  (0.0 – 21.3)  1.4±2.4  (0.0 – 5.3)  1.7±2.7  (0 – 6.7)  26.4±33.6  (0 – 79.9) | 59.4±94.9  (0.0 – 295.2)  265.1±259.6  (0.0 – 910.1)  11.1±18.6  (0.0 – 57.3)  5.4±8.0  (0.0 – 5.3)  0.3±0.5  (0 – 1.7)  4.8±0.8  (2.8 – 6.8) |
| **Group 3** | **K^trans^**  **k_ep_**  **v_e_**  **v_p_**  **AUC**  **mrE** | - | 2.6 ±5.0  (0.0 – 10.5)  128.5 ±257.0  (0.0 – 537.4)  0.5 ±1.0  (0.0 – 2.0)  0.8 ±0.9  (0.0 – 2.3)  0.04±0.08  (0 – 0.2)  6.4±1.2  (3.6 – 9.3) | 0.01±0.02  (0.0 – 0.07)  0.0  0.0  0.0  0.0  0.5±0.2  (0.0 – 1-1)  0.01±0.01  (0 – 0.06)  0.0*  0.0* | 0.93±0  0.0  0.0  0.0  0.0  0.0  1.8±0  0  0.02±0.04  (0 – 0.04)  1.8±0.7  (0.9 – 2.3) |
| **Group 4** | **K^trans^**  **k_ep_**  **v_e_**  **v_p_**  **AUC**  **mrE** | - | 1.5±2.6  (0.0 – 3.9)  146.7±250.8  (0.0 – 378.6)  0.3±0.5  (0.0 – 0.7)  0.7±0.6  (0.1 – 1.2)  0.8±0.2  (0 – 2.8)  5.2±1.4  (0 – 17.6) | 1.8±2.5  (0.1 – 3.6)  160.2±369.4  (0.0 – 408.4)  0.8±2.1  (0.0 – 2.2)  2.8±4.2  (0.0 – 5.6) 0.5±0.4 (0.3 – 0.7)  6.7±1.6  (5.1 – 8.3) | 1.2±1.4  (0.0 – 3.5)  21.0±41.9  (0.0 – 87.7)  0.7±1.5  (0.0 – 3.0)  3.0±3.3  (0.0 – 8.3)  0.3±0.2  (0 – 0.6)  4.2±1.1  (2.4 – 6.0) |
| **Caption:** zone 1: vital tumor, zone 2: infiltration zone, zone 3: normal brain, group 1: short survival:  GBM °IV and astrocytoma °III, IDH wildtype; group 2: shorter intermediate survival: GBM °IV, IDH mutated; group 3: longer intermediate survival: astrocytoma °III, IDH mutated; group 4: longer survival: oligodendroglioma °III and °II, IDH mutated. Units: K^trans^ and k_ep_: 10*^-3^/min.; v_e,_ v_p_ and mrE (maximum relative enhancement: %. * values very close to zero | | | | | |
